# Mitochondrial Dysfunction in Endothelial Progenitor Cells: Unraveling Insights from Vascular Endothelial Cells

**DOI:** 10.3390/biology13020070

**Published:** 2024-01-23

**Authors:** Azra Kulovic-Sissawo, Carolina Tocantins, Mariana S. Diniz, Elisa Weiss, Andreas Steiner, Silvija Tokic, Corina T. Madreiter-Sokolowski, Susana P. Pereira, Ursula Hiden

**Affiliations:** 1 Perinatal Research Laboratory, Department of Obstetrics and Gynaecology, Medical University of Graz, Auenbruggerplatz 14, 8036 Graz, Austria; azra.kulovic@medunigraz.at (A.K.-S.); ctsantos@cnc.uc.pt (C.T.); andreas.steiner@stud.medunigraz.at (A.S.); 2 Research Unit Early Life Determinants (ELiD), Medical University of Graz, Auenbruggerplatz 14, 8036 Graz, Austria; 3 CNC-UC—Center for Neuroscience and Cell Biology, University of Coimbra, Rua Larga, 3004-504 Coimbra, Portugal; 4 Center for Innovative Biomedicine and Biotechnology (CIBB), University of Coimbra, 3000-504 Coimbra, Portugal; 5 Doctoral Programme in Experimental Biology and Biomedicine (PDBEB), Institute for Interdisciplinary Research (IIIUC), University of Coimbra, 3004-531 Coimbra, Portugal; 6 Research Unit of Analytical Mass Spectrometry, Cell Biology and Biochemistry of Inborn Errors of Metabolism, Department of Paediatrics and Adolescent Medicine, Medical University of Graz, Auenbruggerplatz 34, 8036 Graz, Austria; 7 Division of Molecular Biology and Biochemistry, Medical University of Graz, Neue Stiftingtalstraße 6, 8010 Graz, Austria; 8 Laboratory of Metabolism and Exercise (LaMetEx), Research Centre in Physical Activity, Health and Leisure (CIAFEL), Laboratory for Integrative and Translational Research in Population Health (ITR), Faculty of Sports, University of Porto, 4200-450 Porto, Portugal

**Keywords:** mitochondrial dysfunction, reactive oxygen species, cardiovascular risk factors, endothelial dysfunction, endothelial progenitor cells, cardiovascular disease, neurodegenerative disorders

## Abstract

**Simple Summary:**

Endothelial cells (ECs) form the inner lining of all blood vessels. This endothelium has vital functions for the body, and endothelial dysfunction is associated with several lifestyle-related diseases, including cardiovascular and neurodegenerative diseases. Therefore, endothelial dysfunction contributes significantly to the global health burden. Mitochondria are the powerhouses of cells and regulate metabolism and cell behavior. The function of ECs is highly dependent on mitochondria. Cardiovascular risk factors (CVRFs), such as obesity, diabetes mellitus (DM), or chronic inflammation, can impair mitochondria and thus ECfunction. Endothelial progenitor cells (EPCs) are a backup for ECscirculating in the bloodstream. They can be recruited from the blood for endothelial repair. After attachment to the vessel wall, EPCs differentiate into ECs. Recent research has shown that, like ECs, EPCs are also sensitive to CVRFs., but the mechanisms of damage, and whether mitochondria play a role, are not yet known. In this review, we describe the role of mitochondria in endothelial dysfunction. Based on recent studies investigating EPCs in diseases and under the influence of CVRFs, we discuss the role of mitochondria in EPC deterioration. Moreover, we address potential therapeutic interventions targeting mitochondrial health to promote endothelial function.

**Abstract:**

Endothelial dysfunction is associated with several lifestyle-related diseases, including cardiovascular and neurodegenerative diseases, and it contributes significantly to the global health burden. Recent research indicates a link between cardiovascular risk factors (CVRFs), excessive production of reactive oxygen species (ROS), mitochondrial impairment, and endothelial dysfunction. Circulating endothelial progenitor cells (EPCs) are recruited into the vessel wall to maintain appropriate endothelial function, repair, and angiogenesis. After attachment, EPCs differentiate into mature endothelial cells (ECs). Like ECs, EPCs are also susceptible to CVRFs, including metabolic dysfunction and chronic inflammation. Therefore, mitochondrial dysfunction of EPCs may have long-term effects on the function of the mature ECs into which EPCs differentiate, particularly in the presence of endothelial damage. However, a link between CVRFs and impaired mitochondrial function in EPCs has hardly been investigated. In this review, we aim to consolidate existing knowledge on the development of mitochondrial and endothelial dysfunction in the vascular endothelium, place it in the context of recent studies investigating the consequences of CVRFs on EPCs, and discuss the role of mitochondrial dysfunction. Thus, we aim to gain a comprehensive understanding of mechanisms involved in EPC deterioration in relation to CVRFs and address potential therapeutic interventions targeting mitochondrial health to promote endothelial function.

## 1. Introduction

Endothelial cells (ECs) cover the lumen of all blood vessels and fulfill various functions that are essential for the body’s homeostasis. For instance, ECs participate in vascular tone regulation, blood clotting, and immune functions. Endothelial dysfunction arises, in particular, under the influence of cardiovascular risk factors (CVRFs), including obesity, physical inactivity, low-grade inflammation, aging, and smoking [1,2,3,4,5,6]. Endothelial dysfunction is a major contributor to a plethora of cardiovascular disorders [7], which are the leading cause of disease burden worldwide [8]. In particular, oxidative stress plays a key role in endothelial dysfunction and cardiovascular disorders [7,9]. Oxidative stress is characterized by an imbalance between the overproduction and accumulation of reactive oxygen species (ROS) and lower antioxidant defense, which can lead to cell damage by altering proteins, lipids, and nucleic acids [10]. ROS can be formed as signaling molecules generated by enzymes of the redox signaling pathway [11], which is induced by a range of stimuli, including pro-inflammatory cytokines and growth factors [12,13]. ROS are predominantly generated as natural by-products in the mitochondrial electron transport chain (ETC) [9]. Oxidative stress, hypoxia, and metabolic derangements lead to excessive ROS production through oxidative phosphorylation due to uncoupled electron transport in the mitochondrial ETC and adenosine triphosphate (ATP) synthesis [9,14]. Hence, mitochondria are a primary hub in ROS production, ROS signaling, and oxidative stress. Any failure of normal mitochondrial function is referred to as mitochondrial dysfunction and is characterized by a loss of efficiency in energy production paralleled by increased ROS generation [15]. Mitochondrial dysfunction is a characteristic of aging [16] and various chronic diseases [17,18,19] and is closely linked to endothelial dysfunction and cardiovascular disease (CVD) development [20].

Within the cardiovascular system, cardiomyocytes, in particular, have a high density of mitochondria to respond to the energetic demands of the cardiac muscle [21]. In contrast, endothelial mitochondria are not as abundant or relevant for energy production, as in EC, 75% of energy is obtained through glycolysis [22]. Thus, the potential role of endothelial mitochondrial dysfunction in endothelial pathophysiology and CVD has been unnoticed for a long time. However, endothelial mitochondria can generate ROS and relevant other metabolic intermediates. During inflammation, hypoxia, or stress, ROS may exceed its physiological levels, disturbing endothelial function and thus promoting the progression of CVD. Recent research highlights the mitochondria’s central and long-underestimated contribution in endothelial dysfunction [21,22].

In addition to mature ECs, which are part of the vascular endothelium, there is a distinct population of circulating endothelial progenitor cells (EPCs). These cells function as a reserve pool of ECs recruited to repair damaged vascular endothelium or engage in angiogenesis when needed. Upon recruitment into the vascular wall, EPCs differentiate into mature ECs, which is why EPCs are also termed endothelial colony-forming cells (ECFCs) [23]. CVRFs not only adversely affect vascular ECs but also circulating EPCs and trigger reduced EPC numbers and decreased proliferative and angiogenic potential in situations of compromised vascular health [24,25,26]. While EPCs play a vital role in maintaining the vascular endothelium, their specific involvement in endothelial dysfunction repair remains largely unexplored, and the potential impact of mitochondria on EPC function has received limited attention. Therefore, this review aims to elucidate the significance of mitochondrial (dys)function in vascular ECs. It also discusses the largely unexplored area of mitochondrial dysfunction in EPCs and its potential implications in the context of endothelial dysfunction and associated chronic diseases.

## 2. Endothelial Function and Dysfunction

ECs play a critical role in the cardiovascular system. ECs form the inner layer of blood vessels, regulate blood flow, function as a semi-permeable barrier between the circulation and surrounding tissues, participate in immune response, regulate blood clotting, and initiate growth and repair of blood vessels, thus ensuring proper vascular function. If dysfunctional, ECs vastly contribute to the development of CVD. The following subsections will introduce the main endothelial functions and alterations leading to a dysfunctional endothelium.

### 2.1. Endothelial Function Is Versatile

Among their multifaceted features, ECs regulate the vascular tone through the release of vasodilatory and vasoconstrictive molecules, which actively modulate blood vessel diameter. The main vasodilatory molecule released by ECs is nitric oxide (NO), generated by the enzyme endothelial nitric oxide synthase (eNOS). In an immediate response, NO stimulates cyclic guanosine monophosphate (cGMP) production through the enzyme soluble guanylyl cyclase (sGC) in vascular smooth muscle cells (VSMCs), leading to relaxation and vasodilatation [27]. Moreover, NO inhibits the proliferation of VSMCs, prevents platelet and leukocyte adhesion, and inhibits the expression of pro-inflammatory cytokines, thus exhibiting vasodilatory and anti-thrombotic features [3,4]. Other endothelial-derived vasodilating factors are prostacyclin (PGI2) and bradykinin (BK). ECs also secrete balanced levels of vasoconstricting factors, such as endothelin-1 (ET-1), prostaglandin H2 (PGH2), thromboxane A2 (TXA2), or angiotensin II (AngII) [3,4,5], serving as vascular tone regulators.

Furthermore, the endothelium represents an adjustable, semi-permeable barrier. Therefore, ECs form special structures, so-called junctions, between neighboring cells. Three types of junctions contribute to the controlled transfer of macromolecules and immune cells, intercellular communication, and paracellular permeability [28,29,30]. Gap junctions (GJs) enable the transport of small molecules between ECs and support intercellular communication, whilst adherence junctions (AJs) and tight junctions (TJs) form structures that determine paracellular permeability. TJs, on the one hand, fulfill a barrier function by controlling permeability for small molecules and ions, whereas AJs, on the other hand, are mainly required for selective transendothelial migration of immune cells [29]. Environmental signals lead to junctional remodeling, thus regulating permeability and the exchange of nutrients and blood cells [28]. In addition to paracellular transport, molecule transport across the endothelium occurs through transcytosis, i.e., the transcellular transport of molecules via vesicles by carrier-mediated active transport through concentration gradient-dependent facilitated transport, or through diffusion [31]. Endothelial barrier function is organ-specific. For instance, in the brain and retina, the endothelial monolayer is tightly connected to maintain a close barrier, even with reduced transcytosis [32]. The essential transport of glucose occurs via facilitated transport through glucose transporters (GLUT), foremost GLUT1 [33]. In contrast, in the liver and kidneys, the endothelium is discontinuous to allow the desired increased exchange of molecules [34].

ECs also actively participate in immune and inflammatory responses as they not only secrete a plethora of cytokines and chemokines but also express specific cell adhesion molecules for immune cells upon activation by inflammatory signals. Thus, ECs mediate the recruitment and transendothelial migration of leukocytes from the circulation to the target tissue [35].

In healthy conditions, ECs act by secreting anti-coagulant factors, such as tissue plasminogen activator (tPA) [4], in an anti-coagulant way to prevent thrombosis and maintain blood fluidity [4,5]. Upon injury, ECs secrete pro-thrombotic factors, including von Willebrand factor (vWF) and plasminogen activator inhibitor-1 (PAI-1), to induce blood clotting [5].

Two other pivotal roles of ECs are vasculogenesis and angiogenesis, which comprise the formation and growth of blood vessels [3,4,36]. The strongest angiogenic trigger is hypoxia, which induces the release of proangiogenic factors, such as vascular endothelial growth factor (VEGF). Upon binding to the VEGF receptor 2 (VEGFR2) on ECs, VEGF activates quiescent ECs and initiates angiogenesis, ensuring the reestablishment of oxygen and nutrients in the tissue [36,37]. However, other bioactive molecules, including growth factors [38], cytokines [39,40], hormones [41], and non-coding RNAs, such as microRNAs (miRNA) and long non-coding RNAs (lncRNA), also regulate angiogenesis [42].

Given the versatility of these multifaceted functions of ECs, the vascular endothelium can emerge as an extensive and dynamic endocrine organ, acting as a vital interface between the circulation and tissues to ensure body homeostasis. By actively participating in immune responses, coagulation processes, and vascular remodeling, ECs maintain vascular health, playing an indispensable role in the cardiovascular system.

### 2.2. Endothelial Dysfunction: The Central Role of Reactive Oxygen Species

The intricate endothelial functions are crucial for ensuring adequate blood flow and the overall well-being of the heart and vessels, as well as the organs supplied. However, when the endothelium loses balance, a cascade of health issues can unfold, particularly in CVD. This endothelial dysfunction is not an isolated event but rather a consequence of a complex interplay involving various CVRFs. These factors, dependent on lifestyle and health conditions, conspire to activate and inflame ECs, setting the stage for health issues. Obesity, poor dietary habits, physical inactivity, type II diabetes mellitus (T2DM), aging, smoking, chronic inflammation, and even microbial infections are some of the underlying causes, as they create a hostile environment characterized by inflammation and oxidative stress [1,2,3,4,5,6].

**Figure 1:**
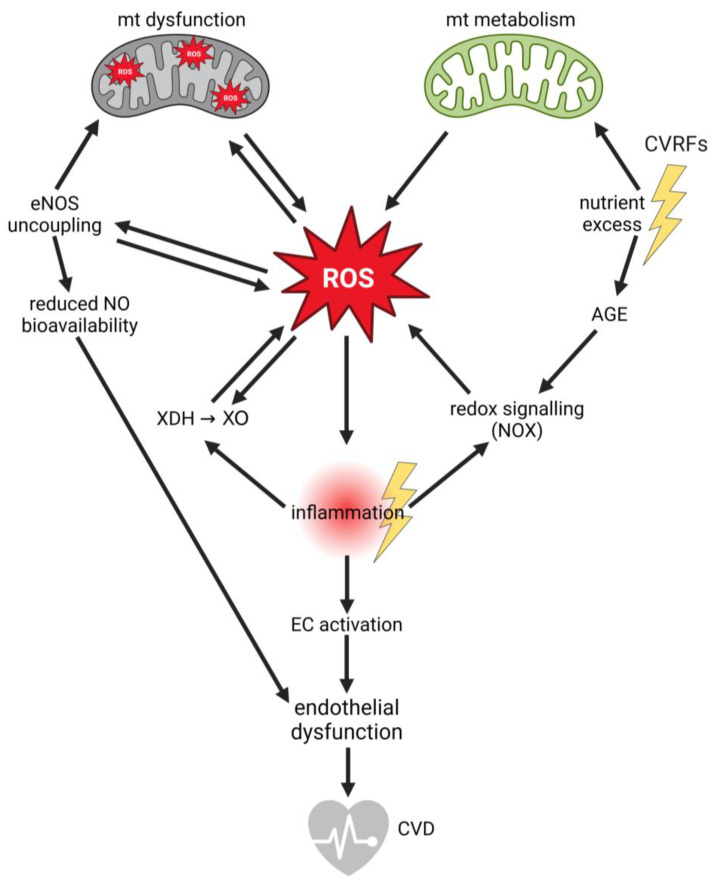
The interplay between metabolism, inflammation, reactive oxygen species, and mitochondrial dysfunction in the development of endothelial dysfunction and cardiovascular disease. Arrows indicate the directionality and stimulation of the respective processes. Influences that represent cardiovascular risk factors (CVRFs), i.e., nutrient excess and inflammation, are marked with yellow flashes. AGE: advanced glycation end products; CVD: cardiovascular disease; EC: endothelial cell; eNOS: endothelial nitric oxide synthase; mt: mitochondrial; NO: nitric oxide; NOX: NADPH oxidases; ROS: reactive oxygen species; XDH: xanthine dehydrogenase; XO: xanthine oxidase. The figure was created using BioRender.com, accessed on 22 January 2024.

Oxidative stress, driven by ROS, is a key player in endothelial (dys)function. Several ROS sources in ECs contribute to oxidative stress generation, which can eventually lead to mitochondrial dysfunction, inflammation, and endothelial dysfunction, as illustrated in Figure 1.

The majority (~90%) of cellular ROS is generated in mitochondria [43,44]. Key contributors are ETC constituents: complex I at the flavin mononucleotide (FMN) site [45], and complex III at the quinol cycle (Q-cycle) [46,47]. Additional ROS-producing enzymes associated with nutrient metabolism and oxidative phosphorylation are succinate dehydrogenase (complex II), glycerol-3-phosphate dehydrogenase (GPD), 2-oxoglutarate dehydrogenase (OGDH), pyruvate dehydrogenase (PDH) complex, proline dehydrogenase (PRODH), dihydroorotate dehydrogenase (DHODH), branched chain keto acid dehydrogenase (BCKDH) complex, acyl-CoA dehydrogenases (very long-chain acyl-CoA dehydrogenase ACDVL; long-chain acyl-CoA dehydrogenase ACADL) [48], electron transfer flavoprotein dehydrogenase (ETFDH), and sulfide quinone reductase (SQR) [49,50], which constitute significant sources of mitochondrial ROS (mtROS). Mitochondrial dysfunction, due to possible damages in the respiratory chain, loss of cytochrome c (CytC), and imbalanced energy demand, is associated with excessive ROS production [51].

In addition to mtROS generated by ETC, redox signaling also contributes to ROS generation. Redox signaling regulates cell growth, differentiation, senescence, apoptosis, and autophagy and is induced by [52,53] pro-inflammatory cytokines and growth factors [12,13,54,55]. By binding to their receptors, reduced nicotinamide adenine dinucleotide phosphate (NADPH) oxidases 1, 2, 4, and 5 (NOX1,2,4,5) become activated and produce ROS as signaling molecules [55,56,57]. NOX enzymes are localized in the plasma membrane and thus contribute to cytosolic ROS [58]. However, NOX4 is also localized in other intracellular compartments, including mitochondria, and it also adds to mtROS [59].

The interaction between glucose molecules and essential cellular components leads to the formation of advanced glycation end products (AGE). These AGE-infused structures become agents of chaos, promoting the release of cytokines, enhancing cell adhesion, and even triggering blood coagulation. The downstream effects are several, influencing everything from angiogenesis to overall endothelial function [60,61,62]. In addition to cytokines and growth factors, the interaction of AGE with their receptor (RAGE) also induces redox signaling by NOX [63].

Another enzyme capable of producing ROS is the purine catabolizing enzyme xanthine dehydrogenase (XDH). Oxidative stress [64,65] or inflammation [66,67] induce post-translational modifications that modify the enzymatic action of XDH to xanthine oxidase (XO) activity, generating superoxide anion (O_2_**·**^−^) [68,69].

Oxidative stress can be self-reinforcing through a process referred to as eNOS uncoupling [70]. Uncoupled eNOS increasingly forms superoxide instead of NO. Superoxide reacts with NO, which is still formed by eNOS at lower levels, to generate peroxynitrite anion (ONOO-) [71]. In mitochondria, peroxynitrite can overwhelm mitochondrial scavenging and repair systems for peroxynitrite-dependent oxidative modifications and, thus, impair mitochondrial energy and calcium (Ca^2+^) homeostasis and membrane permeability. This contributes to mitochondrial dysfunction and augmented ROS production, perpetuating a dysfunction cycle. Uncoupling of eNOS hence promotes and reinforces oxidative stress and mitochondrial dysfunction, but, at the same time, it causes a reduction in NO bioavailability, with severe effects on endothelial function [72,73].

Besides mitochondria, the endoplasmic reticulum (ER) is a source of ROS under certain conditions: ER stress triggers unfolded protein response (UPR), which activates protein kinase RNA (PKR)-like ER kinase (PERK), inositol-requiring protein-1 (IRE1), and activating transcription factor-6 (ATF6). These three UPR signal transduction mechanisms can activate inflammatory signaling via various pathways, including nuclear factor kappa B (NFκB) signaling, which also increases ROS production [74]. Moreover, an ER enzyme involved in disulfide bond formation within protein folding, i.e., ER oxidoreductin (ERO1), generates hydrogen peroxide (H_2_O_2_) [75].

In the scope of ROS generators, red blood cells (RBCs) are also considerable contributors [76,77]. The release of ROS is, on the one hand, induced by endogenous factors, including, in particular, the autoxidation of oxyhemoglobin (HbO_2_) formed by oxygen binding to ferrous heme (FeII) [76,77]. It is thereby oxidized to its ferric form (FeIII), generating methemoglobin (metHb) and superoxide anion [78], which, via several mechanisms, lead to the formation of H_2_O_2_, hydroxyl radical (^•^OH), and hydroxyl anion (OH^−^) [76,79,80]. Notably, superoxide anion -also rapidly reacts with NO, generating the highly reactive peroxynitrite, a potent inducer of endothelial injury [81]. In T2DM, RBC-released ROS induce endothelial dysfunction via arginase I [82], with peroxinitrite operating as an arginase stimulator and mediating the malfunction of ECs [83]. Similar findings were reported in mice models [84].

On the other hand, oxidative stress in RBCs can be triggered by exogenous metabolites like superoxide anion, peroxynitrite anion, and H_2_O_2_ from adjacent cells, including endothelial and immune cells [76]. Thus, besides their role in oxygen transportation, RBCs are crucial for redox balance [85,86], and RBC autoxidation is a considerable source of ROS-promoting oxidative stress in the vasculature [76,77].

Oxidative stress is not an isolated phenomenon. It is closely linked to inflammation as increased ROS reinforce inflammation by promoting leukocyte extravasation and by stimulating cytokine production [3,4,5,87,88]. Pro-inflammatory stimuli destabilize the junctions and thus disrupt the endothelial barrier and increase the permeability [89]. Moreover, oxidative stress per se causes a redistribution of junctional molecules and interferes with signaling pathways associated with barrier function regulation [90].

Under physiological conditions, ROS production and maintenance are regulated through an antioxidant system constituting enzymatic and non-enzymatic factors. The most prominent enzymes are superoxide dismutases (SOD1-3), catalase (CAT), glutathi-one peroxidases (GPX1-7), NAD(P)H quinone dehydrogenase 1 (NQO1), heme oxygenases (HOX1-2), thioredoxin (TXN), and sulfiredoxin 1 (SRXN1). The non-enzymatic system includes uric acid, glutathione, vitamins, and plant secondary metabolites (e.g., polyphenols) [91,92]. These enzymes and antioxidants act in concert to balance the equilibrium between ROS production and oxidative stress.

**Figure 2:**
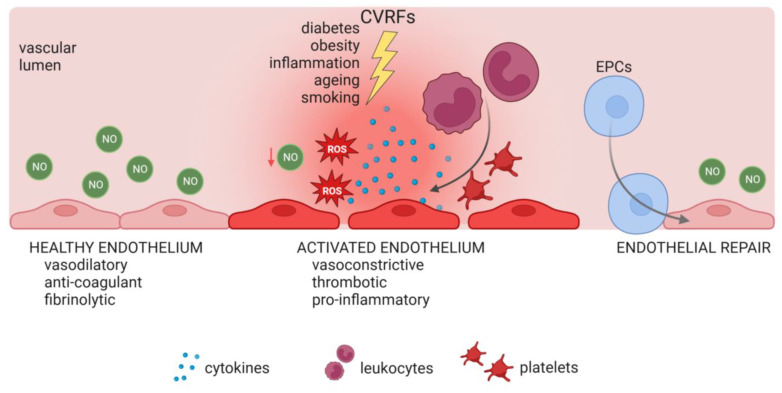
Characteristics of healthy and dysfunctional endothelia and the role of endothelial progenitor cells in repair. Cardiovascular risk factors (CVRFs) disturb normal endothelial function and promote an activated endothelial cell phenotype. A dysfunctional endothelium is accompanied by oxidative stress with increased reactive oxygen species (ROS), inflammation, and reduced nitric oxide (NO) bioavailability. Under healthy conditions, circulating endothelial progenitor cells (EPCs) support, as endothelial colony-forming cells (ECFCs), endothelial repair and recovery. However, it is unclear how CVRFs affect ECFC efficacy and whether the cells remain able to complete repair and restore the endothelium. The figure was created using BioRender.com, accessed on 22 January 2024.

An imbalance in the antioxidant system and ROS production leads to increased oxidative stress, reduced NO bioavailability, and inflammation as the endothelium shifts to an activated, pro-inflammatory, vasoconstrictive, and pro-thrombotic phenotype with increased cytokine and growth factor release, which promote proliferation, migration, and permeability, as well as imbalanced production of vasodilatory vs. vasoconstrictive factors [3,4,5,93] (Figure 2). The intricate interplay between oxidative stress, a pro-inflammatory milieu, and EC activation and dysfunction contributes to endothelial dysfunction and cardiovascular disorders. Notably, the significance of mitochondrial dysfunction as a primary source of ROS in endothelial dysfunction has started to gain recognition.

## 3. Mitochondrial Function in a Healthy Endothelium

Mitochondria are highly dynamic organelles that not only generate energy in form of ATP, but also sense and respond to the surrounding environment. The following subsections will describe the role of mitochondrial dynamics, mitochondrial metabolism, and mtROS in EC function.

### 3.1. Mitochondrial Structure and Dynamics in Endothelial Cells

Mitochondria, essential powerhouses within cells, possess a distinct structure characterized by outer and inner membranes that enclose the mitochondrial matrix [94]. Mitochondrial function is highly dependent on the ETC system, a compilation of proteins intricately associated with the inner membrane comprising four distinct enzymatic complexes (I–IV) [95]. The electron transport is coupled to proton ejection from the mitochondrial matrix into the intermembrane space in every complex except for complex II [95]. Proton ejection generates an electrochemical gradient, creating a proton-motive force to phosphorylate adenosine diphosphate (ADP) into adenosine triphosphate (ATP) through ATP synthase [96,97]. In the intact endothelium, healthy mitochondria appear to have cylindrical structures with an inner mitochondrial membrane with folded cristae enclosing the mitochondrial matrix [98].

The mitochondrial structure is dynamic and balanced between fission and fusion processes, which determine not only mitochondrial shape but also mitochondrial functions, including performance, ROS production, and quality control [99]. The term ‘mitochondrial quality control (MQC) system’ has been established for this network, which tightly balances mitochondrial dynamics, i.e., fission and fusion events and mitophagy [100,101]. Mitochondrial fission is mainly regulated by cytoplasmic dynamin-related protein 1 (DRP1) with the assistance of numerous factors, including mitochondrial fission protein 1 (FIS1), mitochondrial fission factor (MFF), and mitochondrial dynamic proteins (MID49 and MID51) located at the outer membrane [102,103]. Mitochondrial fusion is controlled by membrane proteins mitofusin (MFN) 1 and MFN2, together with optic atrophy protein 1 (OPA1) [102,103]. OPA1 furthermore promotes tight folding of cristae, which increases mitochondrial respiratory efficiency and blunts mitochondrial dysfunction [104]. Mitochondrial dynamics, such as migration and proliferation, are essential for EC function [105], highlighting the central role of mitochondrial morphology for endothelial function.

### 3.2. Mitochondrial Metabolism in Endothelial Cells

The energy metabolism of the vascular endothelium comprises four major metabolic processes: glycolysis, oxidative phosphorylation, fatty acid oxidation (β-oxidation), and glutamine metabolism [106]. Depending on the distinct physiological and pathological stimulations, such as hypoxia and inflammation, cells can adapt their metabolism. This metabolic switch precedes functional changes and disease developments [107,108].

In contrast to neurons and cardiomyocytes, which are highly endowed with mitochondria and perform mitochondrial oxidative phosphorylation and fatty acid oxidation for energy metabolism [109,110], ECs in both macro- and microcirculation depend mainly on glycolysis, which occurs in the cytoplasm [109,110,111,112,113,114,115]. On the one hand, using a less energy-efficient metabolic pathway facilitates oxygen diffusion to surrounding cells by consuming minimal oxygen [116]. On the other hand, using glycolysis can reduce ROS generation [116]. In fact, except for ECs from the blood–brain barrier (BBB) [117], ECs have fewer mitochondria and consume lower amounts of oxygen than other cell types, such as neurons and liver and muscle cells [118,119]. Importantly, mitochondria in ECs have functions other than the generation of ATP, such as biomass generation and signaling [120]. Thus, in a healthy state, ECs are quiescent, mainly relying on glycolysis [111], but this steady cellular metabolism changes during cell activation [111]. During vessel growth and sprouting, fatty acids are important for ECs, being metabolized by mitochondrial fatty acid oxidation [112,121] and thereby producing acetyl-coenzyme A (acetyl-CoA), reduced nicotinamide adenine dinucleotide (NADH) and flavin adenine dinucleotide (FADH_2_) and yielding high amounts of ATP [122]. Also, glutamine metabolism leads into the mitochondrial tricarboxylic acid (TCA) cycle, providing about a third of TCA cycle-derived carbon [123]. However, under normal conditions, ECs do not use fatty acids or glutamine for obtaining energy but mostly for de novo synthesis of nucleotides required for DNA replication and cell proliferation [123,124]. Excess intracellular fatty acids can be stored as cytosolic lipid droplets in ECs [125].

### 3.3. Mitochondrial ROS Homeostasis in Endothelial Cells

Remarkably, even at physiological levels, mtROS and the proteins orchestrating mitochondrial biogenesis play a central role in the regulation of angiogenesis [126]. ROS activate the promoter of the transcription factor hypoxia-inducible factor-1 α (HIF1α) [126,127], which transactivates genes involved in promoting angiogenesis, including VEGF [128], and reinforces VEGFR2 signaling [129]. In contrast, under hypoxic conditions, HIF plays a critical role in maintaining homeostasis. HIF1α and HIF2α promote the activation of the cytochrome c oxidase 4 isoform 2 (COX4I2) subunit gene transcription, resulting in improved electron transfer within the ETC [130]. HIF1α further contributes to decreased complex I activity through induction of the NADH dehydrogenase (ubiquinone) 1 alpha subcomplex, 4-like 2 (*NDUFA4L2*) gene [131]. As mentioned above, mtROS is mainly formed in ETC complex I and III [45,46,47,132,133,134]. Depending on the general cellular conditions, ROS formation can vary between physiological and pathological [132,133,134]. At complex I [132], a high NADH/NAD^+^ ratio results in reduced FMN levels and triggers excessive ROS production. This scenario is induced by damage, ischemia, loss of CytC (apoptosis), and low ATP demand [133,134]. ROS production at complex III mainly happens through auto-oxidation of the Q-cycle intermediate ubisemiquinone [132,133,134].

Moreover, exposure to H_2_O_2_ increases mitochondrial Ca^2+^ concentration in ECs and regulates barrier function maintenance and eNOS activity [22,135,136]. Indeed, NO plays a key role in mitochondria and can inhibit mitochondrial respiratory chain complex I (through S-nitrosylation) and complex IV, modulating EC respiration and ATP production [21]. Dysregulation of this mechanism has also been associated with mitochondrial oxidative stress [21]. Consequently, despite their modest presence within EC, mitochondria harbor the latent potential to exceed physiologic ROS formation, with pathologic ROS levels exerting notable disruptions in endothelial function.

## 4. Unveiling Endothelial Mitochondrial Dysfunction in Pathophysiology

Mitochondrial dysfunction is characterized by a loss of efficiency in the ETC, resulting in reduced synthesis of high-energy molecules, such as ATP [137], increased ROS generation, and oxidative stress [15]. Mitochondrial dysfunction is associated with aging as well as many chronic diseases, including CVD, neurodegenerative disorders (NDDs), metabolic diseases, and chronic infections [16,17,18,19,20,100,138,139,140,141]. The following subsections will describe the mechanisms of dysfunction in ECs and the mitochondrial contribution to CVD, NDDs, and DM.

### 4.1. Mitochondrial Dysfunction in Endothelial Cells

At physiological levels, ROS act as signaling molecules and are beneficial for mitochondria. However, when in excess, ROS are harmful, altering biomolecules and impairing mitochondrial function [142], highlighting the importance of tight mitochondrial regulation of ROS generation. One origin of excess mtROS is damaged mitochondria, which are prone to shifting mitochondrial dynamics to fission, resulting in an overload of mitochondrial fragments. Therefore, a highly efficient mechanism of removing damaged mitochondria exists, i.e., mitophagy, to maintain mitochondrial health, which is of particular value for the cardiovascular system [143,144].

The mitochondrial structure, related to fission and fusion processes, plays a vital role in maintaining the fine-tuning of mitochondrial dynamics and cellular function [145]. In line with this, mitochondrial structural damage has been identified in the context of endothelial dysfunction. For instance, throughout aging, human umbilical vein ECs (HUVECs) present degenerated cristae and swollen regions, along with decreased mitochondrial membrane potential (MMP) and loss of fusion and fission events [146]. Treatment with high glucose and palmitate induces structural changes in rat aortic EC mitochondria, and reduced mitochondrial size is associated with elevated ROS levels and augmented cellular levels of superoxide anion and cytoplasmic H_2_O_2_. This increased oxidative stress is accompanied by a loss of MMP [147]. Furthermore, ECs reveal pronounced alterations, on the one hand, in mitochondrial dynamics, with increased mitochondrial fission (increased FIS1 and phosphorylated-DRP1/DRP1 ratio) and decreased MFN2. On the other hand, ECs differ in apoptosis, with increased expression of cleaved caspase 3 and caspase 9, CytC release, decreased B-cell lymphoma 2 (BCL2), and increased BCL2-like protein 4 (BAX) levels [147]. These data highlight the link between oxidative stress, altered mitochondrial dynamics, mitochondrial dysfunction, and impaired ECs. Mitochondria from HUVEC subjected to high-glucose treatment show an opening of the mitochondrial permeability transition pore (mPTP) and CytC release. These effects are inhibited by overexpression of uncoupling protein 2 (*UCP2*), a mitochondrial protein able to uncouple the oxidative phosphorylation from ATP synthesis by regulating MMP, modulating ROS generation, and contributing to increased NO levels [148]. UCP2 is often upregulated as an adaptive cellular response to demanding environments, and it has a protective role in high-salt-induced injury in ECs by regulating autophagy. Moreover, *UCP2* overexpression results in a higher number of mitochondria and the upregulation of Parkin (PARK2), a critical protein involved in mitophagy [149].

An inflammatory environment mimicked by stimulation with tumor necrosis factor (TNF)α in primary rat aortic ECs resulted in augmented mitochondrial fission with increased NF-κB activation [150]. This response was found to be mediated by Drp1 [150], and, indeed, pharmacological inhibition of mitochondrial fission with mitochondrial division inhibitor 1 (Mdivi-1) improved endothelial function in these cells [147,150].

But, there is another link between mitochondria and inflammation. Deficiency of isocitrate dehydrogenase NADP^+^ 2 (IDH2), a TCA cycle enzyme, is associated with increased endothelial inflammation in HUVEC [151] and contributes to enhanced levels of cytokine transcripts, such as TNFα and interleukin (IL) 1β, coincidently with activated p66shc (SHC adaptor protein 1) [151], a protein known to promote oxidative stress in ECs. Furthermore, Idh2 downregulation and increased activation of p66shc in mouse umbilical vein ECs lead to changes in the abundance of ETC complexes, which result in decreased oxygen consumption [151], demonstrating a link between p66shc and mitochondrial endothelial dysfunction. In fact, this damaging role of p66shc is regulated by sirtuin 1 lysine deacetylase (Sirt1) acetylation [152].

Oxidative stress overload also adversely affects mitochondrial DNA (mtDNA) [142]. In general, circular mtDNA is more prone to ROS-induced damage and mutation not only due to the close proximity to one of the ROS sources, i.e., mtROS, but also because of the lack of additional protection from histones compared to genomic DNA [142]. It has been previously described that EC exposed to ROS undergo mtDNA damage [153], which alters mitochondrial gene and protein expression, impairs mitochondrial function, and contributes to vascular disease development [153].

Impaired mitochondria activate innate immune pathways with the release of mtDNA [154] recognized as damage-associated molecular patterns (DAMPs) by pattern recognition receptors (PRR) [155]. Thereby, the nod-like receptor family pyrin domain-containing 3 (NLRP3) inflammasome is triggered. The NLRP3 inflammasome is a protein complex in the cytoplasm that mediates an innate immune response and detects microbial motifs and endogenous danger signals. NLRP3 induction leads to caspase 1 activation and the release of pro-inflammatory molecules, including cytokines IL1β and IL18, and potentially leads to cell death [156,157,158].

**Figure 3:**
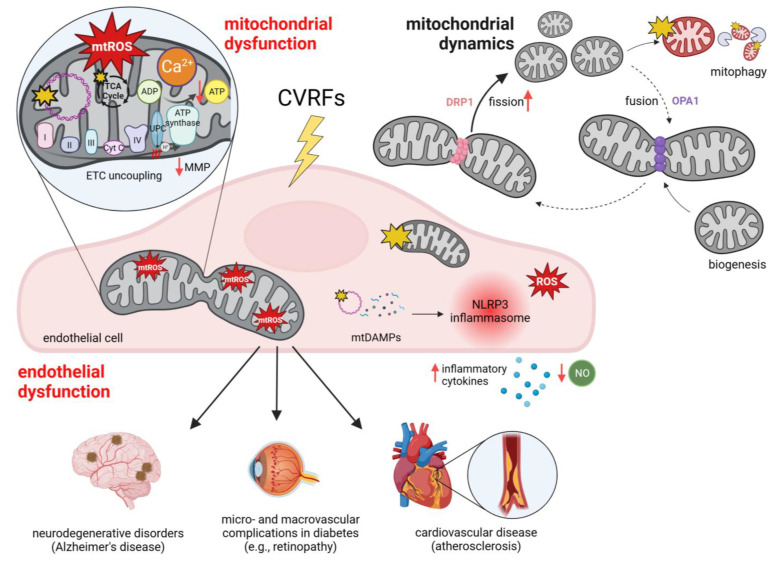
Mitochondria play a decisive role in shaping healthy vs. dysfunctional endothelial phenotypes. Cardiovascular risk factors (CVRFs) trigger detrimental mitochondrial impairment and dysfunction. In this context, impaired or damaged mitochondria discharge reactive oxygen species (mtROS) and mitochondrial-damage-associated molecular patterns (mtDAMPs) into the cytoplasm, which are degraded in the NLRP3 inflammasome. Mitochondrial dynamics shift towards increased fission. Mitophagy, a cellular process that involves the selective removal of damaged or dysfunctional mitochondria, emerges as a guardian of endothelial homeostasis. This process takes on the role of an athero-protective sentinel, as it systematically rids the endothelium of compromised mitochondria, thus safeguarding against the progression of atherosclerosis. Disruption of mitochondrial function and dynamics can pave the way for the onset of endothelial dysfunction and diseases. The figure was created using BioRender.com, accessed on 22 January 2024.

Thus, mitochondrial and endothelial dysfunction are tightly related. Altered and damaging cellular environments, including increased levels of glucose [147,148], palmitate [147], and inflammatory cytokines [150], contribute to endothelial dysfunction, with particular implications for mitochondria-controlled mechanisms. Hyperglycemia, hyperlipidemia, and inflammation represent major CVRFs that ultimately lead to an increased risk for CVD or NDDs (Figure 3). Therefore, tackling the mechanisms involved in mitochondrial dysfunction in ECs can provide critical insights into the development and progression of CVD.

### 4.2. Endothelial Mitochondrial Dysfunction in Atherosclerosis: A Catalyst for Cardiovascular Diseases

Atherosclerosis is a chronic inflammatory condition and a common precursor of CVD [159]. It is characterized by lipid, mainly cholesterol, and fibrin accumulation in the form of atheroma plaques, accompanied by calcification, endothelial activation, and an inflammatory response within arterial walls [160]. One of the major driving forces of the inflammatory response in ECs is low-density lipoprotein (LDL) in its oxidized form (oxLDL), promoting plaque formation [161,162,163]. OxLDL binds to the injured vascular endothelium, attracts immune cells, enhances their adhesion, and thereby initiates an immune response [162].

Mitochondrial dysfunction in atherosclerosis was extensively studied in SMCs [164,165] and immune cells, including macrophages [166,167]. Only recently has the significance of mitochondrial damage in ECs been recognized as a pivotal factor contributing to the derangement of the endothelium in atherosclerosis. Consequently, this recognition bears significant new implications for the understanding of CVD development [20,73,168]. The crucial role of properly functioning endothelial mitochondria is highlighted by several publications extensively reviewing its function in the onset and advancement of atherosclerosis [142,145,169,170,171]. Moreover, the pivotal role of ECs and the result of a damaged endothelium have been widely studied in regard to atherosclerotic progression [172,173].

In fact, endothelial activation [174], accompanied by reduced NO generation [175], initiates atherosclerosis [160], in particular in arterial segments with turbulent flow [174,176] and low wall shear stress [177]. Disturbed flow triggers changes in mitochondrial morphology by stimulating fission, resulting in increased DRP1 levels, excessively fragmented mitochondria, and mtROS release [176]. In vascular pathologies, including atherosclerosis, endothelial mitochondria show functional disturbances and structural changes within the inner arrangement of the mitochondrial membrane and reduced and disorganized cristae [98]. A recent study underlined the importance of mitochondrial dynamics in regard to atherosclerosis progression by investigating the athero-protective role of Opa1 in ECs from LDL receptor (LDLR)-deficient mice [103]. *OPA1* silencing in HUVECs resulted in reduced endothelial migration and increased oxidative stress, highlighting the role of OPA1 in EC response to laminar flow by reducing oxidative stress [103]. When exposed to disturbed flow, *Opa1* expression was reduced in mouse ECs, indicating that endothelial mitochondria indeed tend to fragment under atherosclerotic conditions [103]. An overview of mitochondrial dynamics can be found at the top right in Figure 3.

Endothelial mitochondrial damage can further be induced by *Porphyromonas gingivalis* (*P. gingivalis*), a pathogen found in atherosclerotic plaques, also elevating mtROS [178] and promoting mitochondrial fragmentation in a DRP1-dependent manner [179]. Mitochondrial impairment in *P. gingivalis*-infected ECs is partially regulated by the rat sarcoma (Ras) homolog family member A/Rho associated coiled-coil containing protein kinase 1 (RhoA/ROCK1) pathway activation, resulting in elevated DRP1 phosphorylation levels at Ser616 and promoting DRP1 mitochondrial translocation [180]. Moreover, mitochondria of infected ECs were characterized by a loss of MMP, lower ATP levels [179], and decreased mtDNA copy numbers [180]. These findings emphasize that damaged mitochondria are prone to shifting mitochondrial dynamics to fission.

Besides regulating mitochondrial dynamics [181,182], DRP1 plays an important role in oxLDL-induced endothelial damage, supporting the development of atherosclerosis [183]. The inhibition of this protein by Mdivi-1, studied both in vivo in apolipoprotein E (ApoE)-/- mice [184] and in vitro in HUVECs [185], resulted in athero-protective effects, which suggests its potential as a therapeutic target for multiple CVDs, including atherosclerosis [182,186]. Moreover, oxLDL directly affects the NLRP3 inflammasome in ECs [158,187]. In in vivo studies utilizing endothelial-specific NLRP3 mutant mice, a notable reduction in atherosclerosis severity was observed [187]. The attenuated disease progression was suggested to be due to a lower ROS generation, thus decreasing apoptotic cell death rates [187].

Although the mechanism behind endothelial mechano-transduction remains elusive, recent studies reported oxidative phosphorylation driving mitochondrial ATP generation upon shear stress [188]. Vascular ECs exposed to flow transduce shear stress into mitochondrial ATP synthesis, activating Ca^2+^ influx via purinoceptors, i.e., purinergic receptors [189], with mitochondria regulating Ca^2+^ homeostasis [190]. Elevated intracellular Ca^2+^ levels stimulate NO generation and, therefore, induce flow-dependent vessel relaxation [191]. Thus, changes in shear stress are associated with cardiovascular disorders, i.e., atherosclerosis [192,193,194]. Ca^2+^ overload initiates the opening of mPTP, causing tissue damage, including ischemia-reperfusion injury [195]. Recently, it was found that the expression of endothelial mitochondrial Ca^2+^ uniporter (MCU) complex in HUVECs is modulated by shear stress both on gene expression and protein levels, with the most prominent change in mitochondrial Ca^2+^ uniporter regulator 1 (MCUR1) expression (downregulation) under atheroprone, i.e., disturbed, flow [196]. It is suggested that MCUR1 levels regulate the sensitivity of mPTP to mitochondrial Ca^2+^ concentration [196].

The effect of shear stress on endothelial mitochondria depends on shear stress properties [197,198]. On the one hand, laminar shear stress promotes an anti-inflammatory phenotype [199,200] and positively influences endothelial mitochondria [201,202], and, on the other hand, oscillatory shear stress shows pro-inflammatory characteristics in ECs [203]. Oscillatory shear stress causes mitochondrial dysfunction, producing excessive ROS and inflammation in vascular ECs, followed by mitochondrial-induced inflammation [197]. It promotes an inflammatory environment [204] and directly influences plaque formation and stability [205]. In line with these findings, oscillatory shear stress enhances fission but does not support mitophagy in mouse aortic ECs [203]. Enlarged and swollen mitochondria with damaged membranes, fewer cristae, and an abnormal internal arrangement were observed in the ECs of human atherosclerotic plaques through transmission electron microscopy [98].

### 4.3. Endothelial Mitochondrial Dysfunction in Diabetes Mellitus

Hyperglycemia is a main characteristic of DM, and it is considered a major contributor to endothelial dysfunction, a detrimental event in the pathogenesis of DM-associated micro- and macro-vasculopathies [206]. High intracellular glucose increases ROS levels in ECs, ultimately leading to cell and tissue injury [207]. As ECs rely mainly on glycolysis for their energy source, mitochondria are essential for Ca^2+^ homeostasis and ROS generation. Overproduction of ROS by the mitochondrial ETC caused by hyperglycemia affects various aspects of mitochondrial function, as discussed in the Section 3.3 and Section 4.1. Hyperglycemia-induced endothelial mitochondrial dysfunction ultimately leads to mitochondria-dependent apoptosis [208].

In fact, mitochondrial fragmentation has been identified in ECs isolated from the arm vein of diabetic patients [209] and in retinal and coronary ECs of diabetic rodents [210,211]. These changes in diabetic patients and mice, which correlated with increased FIS1 and DRP1 levels, respectively [209,211], were also observed in aortic ECs cultured under hyperglycemic conditions [209]. Diabetic retinopathy is also associated with disturbed mitochondrial dynamics in human retinal ECs, where the acetylation of MFN2 protein plays a role [212]. However, hyperglycemia also affects other mitochondrial aspects, such as mtDNA repair mechanisms, which are impaired in hyperglycemia. Moreover, downregulation of the lncRNA *lncCytB* is involved in mitochondrial genomic stability and is reduced in streptozotocin (STZ)-induced diabetic mice and human donors with retinopathy [213].

In addition to these isolated effects of DM on the endothelium, hyperglycemia exacerbates mitochondrial dysfunction in ECs in CVD. Mitochondrial fragmentation occurs in hemorrhagic transformation after middle cerebral artery occlusion, but only under conditions of hyperglycemia, i.e., in STZ-induced diabetic mice [214]. In line with these findings, mtROS production is impaired in saphenous veins of coronary artery disease (CAD) when patients are also diabetic [215]. Thus, the endothelial dysfunction induced by hyperglycemic insults in DM multiplies the patient’s cardiovascular risk.

### 4.4. Endothelial Mitochondrial Dysfunction in Neurodegenerative Disorders

Several NDDs are characterized by endothelial dysfunction [216,217,218,219]. Moreover, the risk for dementia is increased by CVRFs, such as obesity, physical inactivity, and smoking [220]. Notably, endothelial mitochondrial dysfunction was associated with the development and progression of several NDDs [19,100,138,218].

The blood brain barrier (BBB) poses a significant challenge in the context of endothelial dysfunction in NDDs, which mediates brain homeostasis [221] and consists of ECs, mural cells, including pericytes and VSMC, and astrocytes [216]. Brain microvascular ECs (BMECs) are directly in contact with circulating factors [221] and, due to their unique features, have a decisive role in maintaining the BBB. Highly developed TJs [222] ensure low BBB permeability and high mitochondria content [138,223,224]. Moreover, BMECs are special regarding their mitochondria. Already in 1977, a distinct difference in endothelial mitochondria abundance, dependent on their properties, was described in rats [224]. The endothelial cytoplasmic volume of the BBB comprises 8–11% of mitochondria, whilst capillary ECs from non-BBB regions have fewer mitochondria, occupying only 2–5% of the cytoplasm [224]. This implicates a higher metabolic activity and capacity and highlights a particular role of mitochondria in the physiology and pathology of ECs from the BBB [224].

In fact, the dominating role of mitochondrial oxidative stress in BMECs and its contribution to BBB damage was recently reviewed by Wang et al. [100]. Mitochondrial ROS [225,226] and oxidized mtDNA [227,228], together with CytC [229], n-formyl peptide [230], and cardiolipins [231] released in the cytoplasm, are recognized as DAMPs and trigger inflammatory responses in BMECs [100,138,232]. The NLRP3 inflammasome is activated by mtROS or mtDNA [227] or through binding to the CD36 membrane receptor, which further activates NF-κB [138]. As an inflammatory response, ECs express cellular adhesion molecules (CAMs), including vascular and intracellular CAMs (VCAM and ICAM), which also stimulate the NLRP3 inflammasome to release pro-inflammatory cytokines, causing BBB injury [138]. The mtDAMP-induced inflammatory response in cerebral ECs (CECs) was also extensively reviewed [138,232]. In addition, lipopolysaccharide (LPS) can efficiently contribute to BBB leakage by triggering an inflammatory response [233] and also by inducing mitochondrial dysfunction [234]. LPS impairs mitochondrial oxidative phosphorylation and reduces mitochondrial function in CECs [234]. Furthermore, by inhibiting oxidative phosphorylation, ECs suffer from TJ disruption [234] and high oxidative stress, promoting mitochondrial fragmentation due to Drp1 activation, which increases BBB permeability [235]. The important role of cerebral endothelial mitochondria for BBB integrity was also shown in vivo through pharmacological mitochondrial inhibition [234]. A disrupted BBB can exacerbate the deposition of disease-specific toxic substances, including amyloid β (Aβ), α-synuclein, fibrin, neurotoxins, and pathogens, with mitochondria being involved in multiple pathological processes leading to unfavorable BBB changes [100].

Alzheimer’s disease (AD) is an NDD that progresses with age; it has the strongest causality for dementia. It is characterized by Aβ accumulation, which leads to plaque formation [236]. Mitochondrial dysfunction has also been proposed as the potential primary cause of AD [237].

In fact, among all cellular organelles, mitochondria are most susceptible to Aβ-induced dysfunction [238]. Exposure of mouse brain capillary ECs to Aβ causes increased oxidative phosphorylation, cellular respiration characterized by accelerated oxygen consumption, and mitochondrial superoxide anion generation, potentially generating oxidative damage [239]. All of these changes are accompanied by elevated mitochondrial Ca^2+^ concentration, with the Ca^2+^ influx regulated by multiple pathways stimulating ROS production and, consequently, mitochondrial dysfunction [239]. Complexing Ca^2+^ with EDTA not only abolished mitochondrial activity dysregulation but prevented morphological changes (superoxide anion-induced fragmentation) and apoptotic cell death, indicating the cytotoxic properties of mitochondrial Ca^2+^-overload [239]. In addition, ECs exposed to Aβ peptides had elevated ROS levels, further contributing to BBB damage [240]. Moreover, Aβ peptides (unmodified, isomerized, and phosphorylated) diversely impact mitochondrial function in vitro, with isomerized Aβ causing the most adverse outcomes: high oxidative stress, cytotoxicity, and increased mitochondrial potential and respiration. This indicates that post-translational Aβ modifications affect endothelial BBB cells [240]. Interestingly, the long-lasting destructive impact of Aβ on mitochondrial respiration capacity is strongest under hypoglycemia in primary human brain ECs, elucidating the underlying mechanism cohering dysglycemia and AD in DM [241]. In Aβ-challenged CECs, H_2_O_2_ synthesis is upregulated together with mitochondrial membrane depolarization [242]. Aβ uptake in endothelial mitochondria is hindered by Coenzyme Q10 (CoQ10), an antioxidant lipophilic coenzyme showing cytoprotective properties [243]. The detrimental impact of Aβ on mitochondria and BBB was also described in humans [244]. Notably, human cerebral microvasculature is characterized by mitochondrial loss in AD [245]. The first in vivo study with transgenic mice investigating mitochondrial abnormalities occurring close to Aβ plaques was published in 2013 [246], which demonstrated that mitochondria proximal to dense Aβ plaques reveal structural and functional abnormalities, including reduced MMP, swollen and dystrophic morphology, and increased mitochondrial loss and fragmentation [246].

Although AD is probably the most prominent and best-studied example of the relationship between mitochondrial dysfunction in ECs and NDDs, it is not a unique phenomenon. Vascular dementia (VD), for example, is caused by CVRFs and is associated with endothelial dysfunction and cardiovascular problems throughout the body. Also, in VD, an implication of mitochondrial dysfunction in ECs is suggested [218]. These findings emphasize the contribution of endothelial mitochondrial dysfunction to the development and progression of NDDs, and targeting mitochondria in this regard is of relevant therapeutic potential.

## 5. Endothelial Progenitor Cells in Health and Disease

Due to the limited regenerative potential of mature vascular ECs, circulating EPCs, which mainly derive from hematopoietic stem cells in the bone marrow [247], can be recruited to support endothelial recovery during vascular growth and repair [24]. In vitro, two main types of EPCs are classified. ‘Early’ EPCs emerge soon after isolation, show a spindle-shaped morphology, proliferate slowly, and have an in vitro life span of only about one month. Early EPCs support the existing endothelium in a paracrine way [23,248]. ‘Late’ EPCs, i.e., ECFCs, are progenitor-derived cells that grow out in culture after several days and form colonies of mature ECs with a cobblestone morphology. ECFCs can form de novo vessels in vitro and in vivo [23,248]. Regardless of completed differentiation, ECFCs still exhibit progenitor cell features. Despite sharing the same phenotype and morphology with mature ECs, e.g., HUVECs, ECFCs not only proliferate faster but also react more sensitive towards angiogenic factors, highlighting their importance in neovascularization and repair mechanisms [249] (Figure 2). Additionally, ECFCs are characterized by high clonogenic potential (colony-forming ability). Differentiated to mature ECs, ECFCs express endothelial markers including CD31, vWF, vascular endothelial (VE)-Cadherin (CD144), CD146, and VEGFR2, and are negative for the leukocyte and monocyte markers CD45 and CD14. Expression of CD34, a marker for vascular EPCs, diminishes throughout in vitro culture [23,250,251]. ECFCs have the ability to home to ischemic tissue and initiate neovascularization [252]. The angiogenic capacity of ECFCs is facilitated by their ability to form new vessels and to release paracrine factors that promote and support vascular repair [250].

ECFCs can be isolated from peripheral or umbilical cordblood (UCB) by culturing mononuclear cells under endothelial-specific conditions [23,251,252]. The cell number is about 15-fold higher in UCB compared to adult peripheral blood, with neonatal ECFCs also showing faster outgrowth [253]. Due to their minimally invasive isolation method, ECFCs enable personalized patient-related studies on endothelial function and dysfunction [254].

### 5.1. Endothelial Progenitor Cells and Cardiovascular Risk Factors: Implications for Cardiovascular Disease and Diabetes

Similar to mature vascular ECs, EPCs are susceptible to CVRFs. The number EPC is reduced in peripheral blood in type I and type II DM and exhibit functional abnormalities [255,256], which worsen throughout the course of DM [255]. Moreover, EPCs from diabetic patients differ regarding in vitro cultivation. For instance, isolated ECFCs from T2DM patients show impaired colony outgrowth, less tube formation, decreased proliferation, migration, and impaired in vivo neovascularization (the latter was shown in an animal model) [25,257]. Notably, improved glycemic control also positively impacts EPC numbers and improves the function of differentiated ECFCs [256,258]. Furthermore, the number of EPCs inversely correlates with body mass index (BMI) [259,260] and levels of insulin, leptin, and C-reactive protein (CRP) [260], with ECFCs originating from obese patients showing a slower proliferation rate [260].

**Figure 4:**
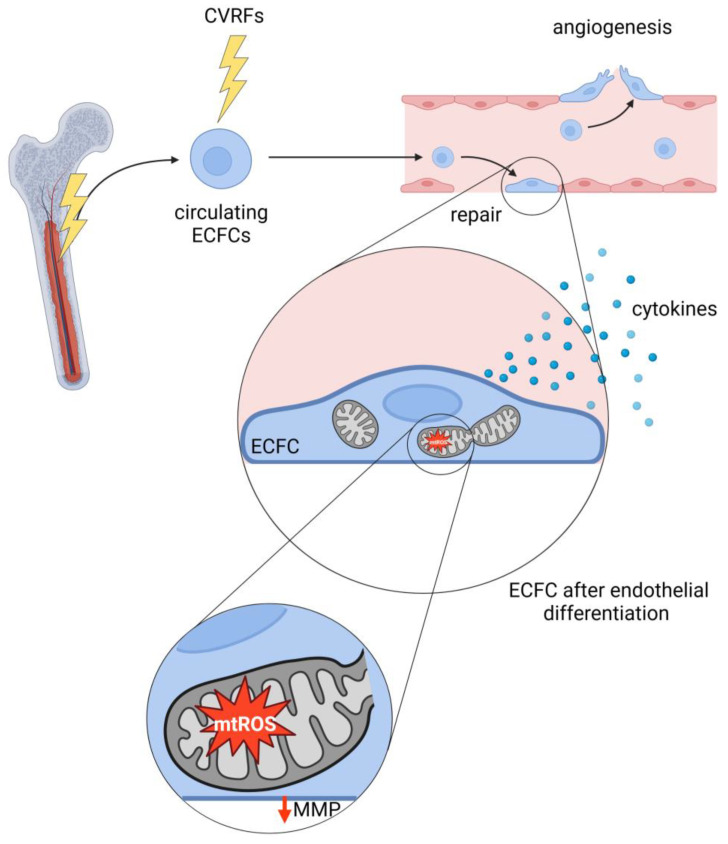
Exposure of endothelial progenitor cells to cardiovascular risk factors disturbs mitochondrial function in the differentiated endothelial cells. Exposure of circulating endothelial progenitor cells (EPCs) and progenitor cells in the bone marrow to cardiovascular risk factors (CVRFs) modulates their mitochondrial function in the long term. Thus, after recruiting the progenitors to the vascular wall, the differentiated endothelial cells remain with dysfunctional mitochondria, elevated reactive oxygen species (ROS) production, reduced mitochondrial membrane potential (MMP), and increased cytokine release. The figure was created using BioRender.com, accessed on 22 January 2024.

These findings indicate that CVRFs have long term effects on circulating EPCs, or even their stem cell precursors (Figure 4). The underlying mechanisms may include epigenetic changes [261,262] as well as covalent modifications of cell components, i.e., oxidative damage of biomolecules, including proteins and lipids, which may lead to potentially irreversible and adverse consequences [263]. Additionally, oxidative DNA damage can induce either permanent genetic or epigenetic changes [264], that might be passed on daughter cells through cell division.

### 5.2. Endothelial Progenitor Cells and Cardiovascular Risk Factors in Pregnancy: Programming of Future Health

In pregnancy, maternal CVRFs may also act on the fetus and affect fetal UCB-derived EPCs. For instance, maternal metabolic state affects EPC function and number. Moreover, maternal pre-pregnancy BMI highjacks the number of fetal UCB-EPCs [265]. Additionally, we have previously demonstrated that during pregnancy, a higher fasting blood glucose within a healthy, non-diabetic range is associated with delayed colony outgrowth of fetal ECFCs [266]. However, there are inconsistencies in the literature regarding the effect of gestational diabetes mellitus (GDM) on fetal EPCs [267]. Several studies reported decreased ECFC colonies with impaired migration and tube formation, accompanied by enhanced cellular senescence and reduced proliferation [26,268]. Others, however, revealed higher proliferation of GDM-derived ECFCs, although with preserved reduced network-formation capacity [269]. In addition, similar outcomes were obtained in fetal EPCs derived from pregnancies complicated by preeclampsia [267]. Findings, such as the developmental programming concept and the Developmental Origins of Health and Disease (DOHaD) paradigm, that describe future susceptibility to disease based on prenatal influences contribute to better understanding of CVD programming in utero.

Thus, circulating EPCs are sensitive to CVRFs, and their acquired impairments may persist even after their recruitment and differentiation to ECs. However, the specific involvement of mitochondria in ECFC dysfunction has remained unexplored.

### 5.3. Endothelial Progenitor Cells in Neurodegenerative Disorders

Early in its disease progression, AD is characterized by the appearance of vascular alterations and BBB disruption [216,244,270,271]. In fact, animal studies in rodents have shown that ECFCs-injections have beneficial effects on plaque deposition and memory [272,273]. Human studies also indicate a role of ECFCs in NDDs, however with variable as well as contradictory results, reporting increased [274], unchanged [275,276], or decreased cell numbers [277,278], possibly due to limited cohort sizes. A new study with over 1500 subjects—currently only published as a preprint—shows a correlation between the number of circulating ECFCs and a reduced risk of AD [279].

ECFC mitochondria in AD have not been investigated so far. As mentioned, CVRFs are affecting ECFCs and therefore, lower cardiovascular risk is associated with slower progression, i.e., cognitive decline, in the general population [280]. Due to the fact that CVRFs cause mitochondrial dysfunction in ECFCs, a link between mitochondrial dysfunction in ECFCs and AD, underlining the importance of future research in this field is suggested.

## 6. Role of Mitochondria in Endothelial Progenitor Cell Dysfunction

As ECFC number and function are sensitive to CVRFs, CVRFs could—similarly to vascular, mature ECs—disrupt mitochondrial function in ECFCs. In fact, mitochondria of senescent human ECFCs demonstrate an elongated shape associated with increased oxidative stress, reduced ATP levels, and decreased mitochondrial fission, as observed by lower FIS1 levels. The same senescent phenotype was induced by *FIS1* silencing in young (low-passage) ECFCs, which demonstrate reduced proliferation activity, denoting the role of FIS1 in mitochondrial and endothelial dysfunction in an aging model [281].

Besides the role of mitochondria in ECFC aging, studies show mitochondrial alterations in ECFCs in patients or animal models with endothelial dysfunction. ECFCs of patients suffering from recurrent venous thromboembolic disease, a condition characterized by impaired endothelial function, reveal elevated ROS levels, cytokine release, and abnormalities in the organization of mitochondrial cristae, with no changes in network formation [282]. ECFCs of hypertensive patients with capillary rarefaction show swollen mitochondria with a loss of mitochondrial cristae, molecularly accompanied by increased ROS and NADH levels. Additionally, mitochondrial bioenergetics are impaired, with decreased oxygen consumption rates (OCR) and reduced MMP. These alterations are paralleled by impaired migration and adhesion of ECFCs and less CXCR4/JAK2/SIRT5 signaling, a pathway involved in mitochondrial metabolic function [283]. Similar results were found in ECFCs differentiated from bone-marrow-derived EPCs of an atherosclerotic mouse model evidencing mitochondrial dysfunction, as revealed by increased size with distorted cristae and elevated mitochondrial superoxide anion generation [284]. Altered mitochondrial function has also been observed in ECFCs isolated from patients with CAD, as demonstrated by higher superoxide anion production. ECFCs derived from CAD patients also possess increased network formation on Matrigel besides migratory and proliferative capacities compared to ECFCs from individuals without CAD [285], highlighting the relationship between mitochondrial and endothelial function. Mitochondrial morphology was, however, not investigated in that study. ECFCs of type II diabetic patients show increased mitochondrial fragmentation and dysregulation of proteins involved in mitochondrial dynamics [286]. Furthermore, and as mentioned before, diabetic ECFCs are functionally compromised, with reduced proliferation, tube formation, and weakened survival capacities [25,257]. However, upregulated expression of nuclear factor erythroid 2–related factor 2 (*NRF2*), a transcription factor involved in redox balance, seems to counteract these DM-induced effects in ECFCs from diabetic patients. and in ECFCs differentiated from bone-marrow-derived EPCs of diabetic mice by regulating the transcription of *IDH2* [286]. The relationship between the metabolic state and mitochondrial function of ECFCs is further highlighted by a study using db/db diabetic mice, a model of T2DM. The study investigated mitochondrial function, i.e., MMP, of bone marrow-derived ECFCs in the bone marrow as a source, in the circulation, and in the retina, where ECFCs are potentially recruited to repair and counteract retinopathy. The decreased MMP of diabetic mice ECFCs is paralleled by impaired peroxisome proliferator-activated receptor alpha (PPARα) levels [287]. The link between the action of CVRFs, circulating EPCs, and mitochondrial dysfunction in the differentiated ECFCs is illustrated in Figure 4.

Further evidence highlighting the relationship between metabolism, oxidative stress, and ECFC function comes from studies investigating the effect of hyperlipidemia on ECFCs. Increased Nox-derived ROS production is characteristic of hyperlipidemic rats and was associated with reduced ECFC adhesion and migration [288]. This link between NOX activity, ROS, and reduced ECFC function was also found in hyperlipidemic patients, where NOX2 and NOX4 RNA expression and protein levels are increased in ECFCs, which is associated with reduced ECFC adhesion, migration, and tube formation [289].

Apart from the earlier described detrimental effects that pathologies cause in the mitochondrial function of ECFCs, in vitro experiments also highlight the interplay of mitochondrial function and ROS with ECFC function [290] in physiology. For instance, in vitro experiments have demonstrated that the pulsatile pressure within the blood vessels promotes vascular homing of ECFCs, both by stimulating adhesion and endothelial differentiation. Cyclic stretch, when applied to ECFCs, decreases the content of long-chain fatty acids (LCFAs) and induces the expression of long-chain fatty acyl-CoA synthetase 1 (*ACSL1*), which facilitates the catabolism of LCFAs in mitochondria via fatty acid oxidation and oxidative phosphorylation [290]. Transplantation of ECFCs overexpressing *ACSL1* into a rat carotid artery injury model enhances ECFC adhesion and endothelialization. Furthermore, ROS signaling within the physiological range has positive effects on ECFC function: Action of NOX4, the major ROS-producing enzyme in ECFCs, stimulates angiogenesis in these cells by upregulating pro-angiogenic factors linked with eNOS signaling [291], highlighting the importance of fine-tuning mitochondrial metabolism for ECFC function.

The role of mitochondria in the angiogenesis of rat ECFCs has been further emphasized by the fact that pyruvate kinase M2 (Pkm2), a protein responsible for energy metabolism and mitochondrial morphology, promotes ECFC angiogenesis through modulation of glycolysis, mitochondrial fission, and fusion [292]. Further evidence relating mitochondrial function to angiogenesis of ECFCs comes from a study using very low-density lipoprotein receptor knockout mice as a model of ocular neovascularisation induced by Wnt signaling overactivation. The study revealed that circulating EPCs of this mouse model possess higher MMP, with isolated ECFCs showing increased mitochondrial function and biogenesis, as well as a more active state towards endothelial differentiation [293].

As pointed out in this critical review, recent studies clearly show that despite the relatively low number of mitochondria in ECs, mitochondrial dysfunction and ROS are major contributors to endothelial dysfunction. In regard to EPCs and ECFCs, respectively, there are less data available, but these also suggest that mitochondrial function is essential for ECFC physiology and pathology. In summary, the evidence supports the proposition that mitochondrial dysfunction in ECFCs and ECs is intricately linked to endothelial dysfunction and CVD pathogenesis. However, whether this also applies to NDDs, such as AD, remains to be investigated.

## 7. Mitochondria-Targeted Therapeutic Strategies to Improve Endothelial Function

In recent years, several strategies aiming to restore optimal mitochondrial function have emerged. Notable approaches include using mitochondrial-targeted antioxidants, mitophagy inducers, and mitochondrial biogenesis enhancers. The mitochondrial-targeted antioxidants are compounds specifically targeting mtROS and counteracting oxidative stress. By restoring redox balance, these compounds hold promise for mitigating mitochondrial impairment in ECFCs and ECs, thus ultimately thwarting CVD progression. MtROS overproduction can be hindered, for example, by mitoquinone (MitoQ) [294], a mitochondria-targeting antioxidant accumulating within the organelle and neutralizing oxidative stress [294]. Findings from a randomized controlled trial revealed that acute oral intake of MitoQ restored mitochondrial function and improved endothelial function in patients suffering from peripheral artery disease [295]. Acute and, importantly, chronic intake of MitoQ delivered promising results in elderly adults [296]. In an ex vivo model, exposure of human aortic ECs (HAECs) to plasma from MitoQ-treated adults reduced mtROS, lowered circulating oxLDL levels, and improved endothelial properties [206]. Other promising mitotropic molecules are SkQ1 [297,298,299,300], MitoTEMPO [301], SS-31 [302,303], and AntiOxCIN4 [304,305,306]. As discussed, enhancing mitophagy can prevent the accumulation of dysfunctional mitochondria, preserve cellular health, and improve EPC and EC function. Rapamycin [307,308], urolithin A [309], carbonyl cyanide m-chlorophenyl hydrazone (CCCP) [310], and PTEN-induced kinase 1 (PINK1)/parkin pathway activators [311] were described as potential mitophagy inducers. Another therapeutic strategy is using mitochondrial biogenesis enhancers to facilitate mitochondrial function. That would allow for replenishing the pool of functional mitochondria, bolstering cellular energy production, and combating dysfunction. Resveratrol, PPARγ, Adenosine monophosphate (AMP)-activated protein kinase (AMPK) activators, carnitine, berberine, exercise, and starvation have been described as mitochondrial biogenesis activators [312,313,314,315,316,317,318].

Implementing these strategies carries profound implications for comprehending the pathogenesis of CVD and NDDs and formulating therapeutic approaches. Directing attention toward mitigating mitochondrial dysfunction provides an innovative perspective for addressing the fundamental mechanisms fueling endothelial dysfunction. By reinstating optimal mitochondrial function within EPCs and ECs, the progression of endothelial dysfunction, atherosclerosis, other cardiovascular complications, and NDDs may potentially be abated.

## 8. Conclusions and Future Perspectives

Endothelial dysfunction resulting from the action of CVRFs underlies and contributes to various non-communicable and age-related diseases, including CVD, NDDs, and metabolic diseases. CVD, for instance, has been categorized by the WHO as the disease with the highest mortality worldwide [319]. The relationship between CVRFs, mitochondrial and endothelial dysfunction highlights that a profound understanding of endothelial mitochondrial damage is crucial to improve the prevention and treatment of CVD and NDDs. Until now the role of ECFCs in CVD and NDDs is not yet fully understood. However, the fact that not only ECs but also circulating ECFCs and even their precursors located, for instance, in the bone marrow, are damaged by CVRFs demonstrates the harm that CVRFs exert on the vasculature. Circulating ECFCs would normally be responsible for endothelial repair and recovery. It is, therefore, all the more important to understand the cellular processes of CVD and other non-communicable diseases to develop possible therapies.

Within the framework of the DOHaD concept, an intriguing question surfaces: Can mitochondrial dysfunction be orchestrated by early influential factors in utero? This perspective aligns with the notion that events occurring during critical developmental stages might exert a lasting impact on mitochondrial health, consequently contributing to the trajectory of endothelial, cardiovascular, and neuronal health or susceptibility to disease later in life [320]. This introduces an additional layer of complexity when striving to address CVD. It is imperative to account for the fact that a significant proportion of the proposed strategies to enhance mitochondrial function have not undergone testing during pregnancy, except for exercise [321,322,323,324,325,326] and MitoQ [327,328,329], nor has the particular effect on ECs or EPCs been evaluated.

Future research endeavors should delve deeper into the mechanisms that contribute to mitochondrial dysfunction in EPCs and ECs. Additionally, clinical translation of these strategies requires rigorous testing in preclinical models and human trials to validate their efficacy, safety, and long-term benefits. As our understanding of mitochondrial involvement in endothelial dysfunction deepens, successfully translating these approaches could revolutionize cardiovascular therapeutics, potentially leading to more effective strategies for managing and preventing CVD and NDDs.

## Data Availability

Not applicable.

## Abbreviations

OGDH—2-oxoglutarate dehydrogenase; acetyl-CoA—acetyl-coenzyme A; ATF6—activating transcription factor-6; ADP—adenosine diphosphate; AMP—Adenosine monophosphate; ATP—adenosine triphosphate; AJs—adherence junctions; AGE—advanced glycation end products; RAGE—AGE receptor; AD—Alzheimer’s disease; AMPK—AMP-activated protein kinase; Aβ—amyloid β; AngII—angiotensin II; BCL2—B-cell lymphoma 2; BAX—BCL2-like protein 4; BBB—blood–brain barrier; BMI—body mass index; BK—bradykinin; BMECs—Brain microvascular ECs; BCKDH—branched chain keto acid dehydrogenase; Ca^2+^—calcium; CCCP—carbonyl cyanide m-chlorophenyl hydrazone; CVD—cardiovascular disease; CVRFs—cardiovascular risk factors; CAT—catalase; CAMs—cellular adhesion molecules; CECs—cerebral ECs; CoQ10—Coenzyme Q10; CAD—coronary artery disease; CRP—C-reactive protein; cGMP—cyclic guanosine monophosphate; CytC—cytochrome C; COX4I2—cytochrome c oxidase 4 isoform 2; DAMPs—damage-associated molecular patterns; DOHaD—Developmental Origins of Health and Disease; DM—diabetes mellitus; DHODH—dihydroorotate dehydrogenase; DRP1—dynamin-related protein 1; ETFDH—electron transfer flavoprotein dehydrogenase; ETC—electron transport chain; ER—endoplasmic reticulum; ECs—Endothelial cells; ECFCs—endothelial colony-forming cells; eNOS—endothelial nitric oxide synthase; EPCs—Endothelial progenitor cells; ET-1—endothelin-1; ERO1—ER oxidoreductin; FeIII—ferric iron; FeII—ferrous iron; FADH2—flavin adenine dinucleotide; FMN—flavin mononucleotide; GJs—Gap junctions; GDM—gestational diabetes mellitus; GLUT—glucose transporters; GPX1-7—glutathione peroxidases; GPD—glycerol-3-phosphate dehydrogenase; HOX1-2—heme oxygenases; HUVECs—human umbilical vein ECs; H2O2—hydrogen peroxide; OH^−^—hydroxyl anion; ^•^OH—hydroxyl radical; HIF1a—hypoxia-inducible factor-1 a; IRE1—inositol-requiring protein-1; IL—interleukin; ICAM—intracellular CAM; IDH2—isocitrate dehydrogenase NADP^+^ 2; LDLR—LDL receptor; LPS—lipopolysaccharide; lncRNA—long non-coding RNAs; ACADL—long-chain acyl-CoA dehydrogenase; LCFAs—long-chain fatty acids; ACSL1—long-chain fatty acyl-CoA synthetase 1; LDL—low-density lipoprotein; MFN—membrane proteins mitofusin; metHb—methemoglobin; miRNA—microRNAs; MCU—mitochondrial Ca^2+^ uniporter; MCUR1—mitochondrial Ca^2+^ uniporter regulator 1; Mdivi-1—mitochondrial division inhibitor 1; mtDNA—mitochondrial DNA; MID—mitochondrial dynamic proteins; MFF—mitochondrial fission factor; FIS1—mitochondrial fission protein 1; MMP—mitochondrial membrane potential; mPTP—mitochondrial permeability transition pore; MQC—mitochondrial quality control; mtROS—mitochondrial ROS; MitoQ—mitoquinone; NQO1—NADPH quinone dehydrogenase 1; NDUFA4L2—NADH dehydrogenase ubiquinone 1 alpha subcomplex, 4-like 2; NOX—NADPH oxidase; NDDs—neurodegenerative disorders; NADH—nicotinamide adenine dinucleotide; NADPH—nicotinamide adenine dinucleotide phosphate; NO—nitric oxide; NLRP3—nod-like receptor family pyrin domain-containing 3; NRF2—nuclear factor erythroid 2–related factor 2; NFκB—nuclear factor kappa B; OPA1—optic atrophy protein 1; oxLDL—oxidized LDL; OCR—oxygen consumption rates; HbO2—oxyhemoglobin HbO2; PARK2—Parkin; PRR—pattern recognition receptors; PPAR α/γ—Peroxisome Proliferator-Activated Receptor PPAR Alpha/Gamma; ONOO^−^—peroxynitrite anion; PAI-1—plasminogen activator inhibitor-1; *P. gingivalis*—*Porphyromonas gingivalis*; PRODH—proline dehydrogenase; PGI2—prostacyclin; PGH2—prostaglandin H2; PERK—protein kinase RNA PKR-like ER kinase; PINK1—PTEN-induced kinase 1; PDH—pyruvate dehydrogenase; Pkm2—pyruvate kinase M2; Q-cycle—quinol cycle; RhoA—Ras homolog family member A; Ras—rat sarcoma; ROS—reactive oxygen species; RBCs—red blood cells; ROCK1—Rho associated coiled-coil containing protein kinase 1; Sirt1—sirtuin 1 lysine deacetylase; SMCs—smooth muscle cells; sGC—soluble guanylyl cyclase; STZ—streptozotocin; SRXN1—sulfiredoxin 1; O_2_^•−^—superoxide anion; SOD—superoxide dismutase; TXN—thioredoxin; TXA2—thromboxane A2; TJs—tight junctions; tPA—tissue plasminogen activator; TCA—tricarboxylic acid; TNFα—tumor necrosis factor alpha; T2DM—type II DM; UCB—Umbilical cordblood; UCP2—uncoupling protein 2; UPR—unfolded protein response; VCAM—vascular CAM; VD—Vascular dementia; (VE)-Cadherin—vascular endothelial Cadherin; VEGF—vascular endothelial growth factor; VSMCs—vascular SMCs; VEGFR2—VEGF receptor 2; ACDVL—very long-chain acyl-CoA dehydrogenase; vWF—von Willebrand factor; XDH—xanthine dehydrogenase; XO—xanthine oxidase.
